# Early interventions: from e-health to neurobiology

**DOI:** 10.3402/ejpt.v6.28545

**Published:** 2015-06-11

**Authors:** Miranda Olff, Mirjam Van Zuiden, Anne Bakker

**Affiliations:** 1 Department of Psychiatry, Academic Medical Centre, University of Amsterdam, Amsterdam, The Netherlands; 2 Arq Psychotrauma Expert Group, Diemen, The Netherlands

Eighty percent of adults experience at least one traumatic event throughout their lives and approximately 10% of them subsequently develop a posttraumatic stress disorder (PTSD; e.g., De Vries & Olff, 2009). Trauma exposure, especially when prolonged and in childhood, may have negative health consequences throughout the lifespan, in severe cases leading not only to psychological morbidity but also to increased physical diseases such as cardiac disease, cancer, and early death (Vaccarino et al., 2013; Wahlström, Michélsen, Schulman, Backheden, & Keskinen-Rosenqvist, 2013). Thus, there clearly is a significant public health impact of exposure to trauma and much to gain with effective preventive interventions.

Most trauma-exposed individuals will display acute symptoms of posttraumatic stress, but in most of them these will gradually disappear over time. However, a subgroup of trauma-exposed individuals will continue to develop a trauma-related psychiatric disorder. Some time ago, it was believed that “debriefing” was a good solution, helping people to express their emotions and giving them psycho-education after trauma. However, research soon showed that the classical forms of debriefing were not effective and even worsened symptoms in some (Sijbrandij, Olff, Reitsma, Carlier, & Gersons, 2006). Psychosocial support and psychological first aid are evidence-informed interventions expected to be beneficial (Olff, 2012).

To provide the highly needed easily applicable and cost-effective early interventions, researchers have focused on finding new forms of potential early interventions using both pharmacological (e.g., hydrocortisone [Delahanty et al., 2013], morphine [Mouthaan et al., 2015] or oxytocin [Koch et al., 2014]) and psychological routes, such as innovative dyadic CBT by Brunet, Bousquet Des Groseilliers, Cordova, and Ruzek (2013), whereas Rothbaum et al. (2012) performed the first trial showing early exposure treatment to be effective shortly after trauma. Recently, e-health has been added as a promising agent to deliver preventive interventions (Mouthaan et al., 2013; Olff, 2015).

At the AMC, we are currently testing the efficacy of the neuropeptide oxytocin shortly after trauma (BONDS) and the efficacy of mobile apps to detect and reduce trauma-related symptoms (SAM, SUPPORT Coach).

In the BONDS study, we examine the efficacy of intranasal administration of the neuropeptide oxytocin in the prevention of PTSD (Frijling et al., 2014, Olff et al., 2015). Recently traumatized individuals at increased risk for PTSD due to high levels of acute distress are identified at various emergency departments throughout Amsterdam. Within 10 days posttrauma they commence an 8-day treatment regimen of either intranasal oxytocin or placebo. Follow-up occurs at 1.5, 3, and 6 months after trauma. Results of interim analyses on the first 120 randomized participants regarding PTSD symptoms at 1.5 months after trauma will be presented.

SAM, Smart Assessment on your Mobile, is a brief mobile screener to assess psychological problems and resilience after a traumatic event as well as risk and protective factors (Van der Meer et al., 2014). We investigate the validity and usability, and collect data in different samples of trauma-exposed employees in high-risk professions such as the police. Preliminary results demonstrate SAM's usability and point at high user satisfaction. Our second e-health project concerns the Dutch equivalent of the PTSD Coach: The SUPPORT Coach, a smartphone application designed to support adults who suffer from posttraumatic stress symptoms (PTSS). In an ongoing RCT, we test the effectiveness of the SUPPORT Coach in reducing PTSS and satisfaction with the app in individuals in high-risk professions, such as emergency department personnel and ambulance workers.

This conference was funded by a grant from The Swedish Foundation for Humanities and Social Sciences (F14-1747:1).

## References

[CIT0001] Brunet A, Bousquet Des Groseilliers I, Cordova M, Ruzek J (2013). Randomized controlled trial of a brief dyadic cognitive–behavioral intervention designed to prevent PTSD. European Journal of Psychotraumatology.

[CIT0002] De Vries G. J, Olff M (2009). The lifetime prevalence of traumatic events and posttraumatic stress disorder in the Netherlands. Journal of Traumatic Stress.

[CIT0014] Delahanty D. L, Gabert-Quillen C, Ostrowski S. A, Nugent N. R, Fischer B, Morris A (2013). The efficacy of initial hydrocortisone administration at preventing posttraumatic distress in adult trauma patients: a randomized trial. CNS Spectr.

[CIT0003] Frijling J. L, Van Zuiden M, Koch S. B, Nawijn L, Goslings J. C, Luitse J. S (2014). Efficacy of oxytocin administration early after psychotrauma in preventing the 105 development of PTSD: Study protocol of a randomized controlled trial. BMC Psychiatry.

[CIT0004] Koch S. B, Van Zuiden M, Nawijn L, Frijling J. L, Veltman D. J, Olff M (2014). Intranasal oxytocin as strategy for medication-enhanced psychotherapy of PTSD: Salience processing and fear inhibition processes. Psychoneuroendocrinology.

[CIT0005] Mouthaan J, Sijbrandij M, De Vries G.-J, Reitsma J. B, Van De Schoot R, Goslings J. C (2013). Internet-based early intervention to prevent posttraumatic stress disorder in injury patients: Randomized controlled trial. Journal of Medical Internet Research.

[CIT0006] Mouthaan J, Sijbrandij M, Reitsma J. B, Luitse J. S, Goslings J. C, Gersons B. P (2015). The role of early pharmacotherapy in the development of posttraumatic stress disorder symptoms after traumatic injury: An observational cohort study in consecutive patients. General Hospital Psychiatry.

[CIT0007] Olff M (2012). Bonding after trauma: On the role of social support and the oxytocin system in traumatic stress. European Journal of Psychotraumatology.

[CIT0008] Olff M (2015). Mobile mental health paper. European Journal of Psychotraumatology.

[CIT0015] Olff M, Van Zuiden M, Koch S, Nawijn L, Frijling J, Veltman D (2015). Intranasal oxytocin: miracle cure after trauma?. European Journal of Psychotraumatology.

[CIT0009] Rothbaum B. O, Kearns M. C, Price M, Malcoun E, Davis M, Ressler K. J (2012). Early intervention may prevent the development of posttraumatic stress disorder: A randomized pilot civilian study with modified prolonged exposure. Biological Psychiatry.

[CIT0010] Sijbrandij E. M, Olff M, Reitsma J. B, Carlier I. V. E, Gersons B. P. R (2006). Emotional or educational debriefing after psychological trauma: Randomised controlled trial. The British Journal of Psychiatry.

[CIT0011] Vaccarino V, Goldberg J, Rooks C, Shah A. J, Veledar E, Faber T. L (2013). Post-traumatic stress disorder and incidence of coronary heart disease: A twin study. Journal of the American College of Cardiology.

[CIT0012] Van der Meer C. A. I, Bakker A, Broeksteeg J, Schrieken B, Olff M (2014). Enhancing self-screening for trauma related symptoms through a mobile application: The rationale and design of a validation study.

[CIT0013] Wahlström L, Michélsen H, Schulman A, Backheden H, Keskinen-Rosenqvist R (2013). Longitudinal course of physical and psychological symptoms after a natural disaster. European Journal of Psychotraumatology.

